# Lattice variation upon water adsorption in silica opals measured by *in situ* atomic force microscopy†

**DOI:** 10.1039/d5na00127g

**Published:** 2025-03-21

**Authors:** Francisco Gallego-Gómez, Eider Berganza, Miguel Morales, Álvaro Blanco, Cefe López, Agustina Asenjo, Miriam Jaafar

**Affiliations:** a Instituto de Ciencia de Materiales de Madrid (ICMM), Consejo Superior de Investigaciones Científicas (CSIC) Calle Sor Juana Inés de la Cruz 3 E-28049 Madrid Spain eider.berganza@csic.es a.blanco@csic.es

## Abstract

Colloidal photonic crystals, or artificial opals, derive their unique optical properties from the periodic arrangement of their constituent particles. Central to the functionality of these materials is the precise control over their lattice parameter, which directly determines the photonic bandgap. Adsorption of environmental vapors may have a significant impact on the photonic response, most of which has been attributed to lattice variations. Here we aim to directly measure the arrangement and lattice parameter in opals made of silica spheres by using *in situ* atomic force microscopy (AFM) under changing ambient conditions. The periodicity of the opal structure offers a decisive advantage for such study, enabling the use of correlation tools for image processing. Opals having high mechanical stability (avoiding lattice distortions or sphere detachment during scanning) allow reliable AFM data and good analytical resolution. We show direct evidence of reversible lattice increments upon water adsorption, up to several nanometres in agreement with prior indirect estimates, which is compatible with swelling of the spheres due to filling of silica micropores.

## Abstract 2

This study shows reversible lattice expansion in silica opals due to water adsorption, observed *via in situ* AFM. The nanometer-scale changes, linked to sphere swelling and silica micropore filling, affect their photonic properties.

## Graphical abstract

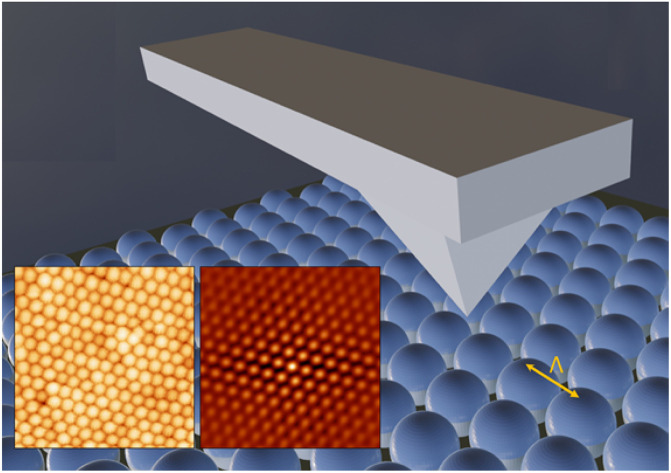

## Introduction

Opals are three-dimensional solid colloidal crystals made of self-assembled spheres, which have been extensively studied in the last few decades and their photonic properties are well understood.^1^ Sub micrometre silica spheres forming a face-centred-cubic (fcc) packing^2^ are commonly used because of the ease of fabrication. Such sphere packings are multiporous structures capable of adsorbing significant amounts of water from the surrounding moisture, especially in the case of hydrophilic, highly microporous Stöber silica spheres.^3^ As periodic dielectric structures, opals exhibit pseudo photonic bandgaps (PBGs) at certain energy ranges, for which the propagation of light is prohibited. PBGs are determined by, principally, the length of the packing lattice and the materials comprised in the structure (both spheres and voids, which involve silica, air and adsorbed water). A clear correlation of the PBG properties with water uptake has been stated.^4,5^

In particular, PBG monitoring under varying ambient conditions has been attempted to infer the concomitant morphological changes within the opal, deducing large, reversible lattice increments upon water adsorption, up to 5% (13 nm) in opals of 335 nm silica spheres.^4^ Such lattice variation, as it implies important structural changes upon water ad/desorption, is relevant to understand photonic crystals and, in general, colloidal arrangements under varying ambient conditions. However, the PBG dependences, not only on the lattice parameter but also on the refractive index distribution along the structure, are complex and thus the correspondence between adsorption-induced PBG changes and lattice variation is not straightforward.^4,6^ Therefore, it is highly desirable to assess lattice changes upon water ad/desorption in a more direct way. AFM is an appropriate tool to characterize *e.g.* interfacial layers^7,8^ or solid–liquid interfaces.^9^ However, apart from the study of growth mechanisms and dynamic scaling of surface width,^10^ the AFM technique has only marginally been performed on opals as a standard structural characterisation tool.^11^ Here we present a systematic AFM study on silica opals of 240 nm diameter (determined by scanning electron microscopy under vacuum conditions), in which the sphere arrangement is *in situ* monitored under varying ambient conditions and the lattice properties are analysed (see Section 1 of ESI†). The high stability of the opal structure^12^ kept the sphere arrangement unaltered under the action of the scanning tip. Besides, the opal order allowed the use of autocorrelation tools to calculate average lattice dimensions with high precision.

## Results and discussion

### A AFM on opals: general considerations experimental

**Fig. 1:**
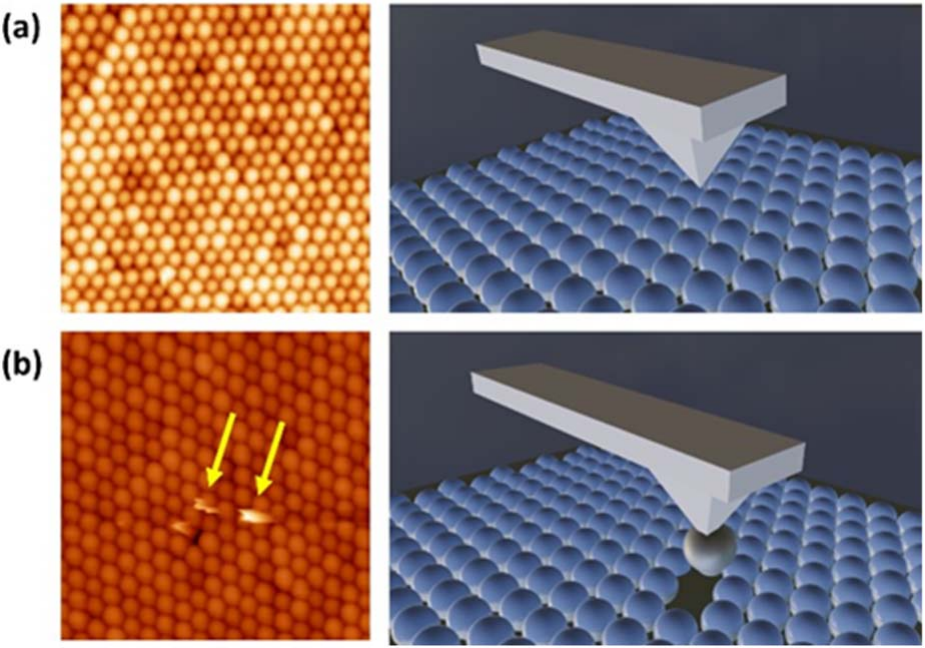
Examples of AFM experiments on a silica opal, typically showing good stability and providing well-defined images (a), and occasional artefacts arising from adhesion between the tip and the spheres, usually in less ordered areas (b).

It is known that AFM implementation on individual particles or particulate systems under standard ambient conditions is greatly complicated by the presence of moisture, which leads to adhesion phenomena between the AFM tip and the sample.^13,14^ This fact usually causes important image distortion, tip contamination, tip-induced diffusion of particles and adulteration of quantitative data. Such drawbacks are present even working in non-contact mode since the water meniscus between the tip and sample might suddenly change, leading to the diffusion of a sphere from the surface to the tip^15^ and it often compels to work under vacuum conditions (which, in general, essentially alters the original situation). In this sense, opals offer a decisive advantage since they exhibit a relatively high cohesion, preventing the deformation of the opal surface during AFM scanning. On one hand, fcc sphere arrangement (the most-compact packing) provides the maximum number of interparticle contacts and thus highest stability.^16^ On the other hand, the presence of moisture itself actually endows the opal with higher mechanical strength as the adsorbed water between the spheres builds bridges exerting capillary cohesion.^12^Fig. 1a represents a typical measurement under normal working conditions (see the Experimental section), showing a flat, uniform opal surface. Particle ripping or structural distortion may occur when a water meniscus is formed between the AFM tip and the opal sample during tapping (Fig. 1b), giving rise to highly noisy scanning lines. However, this is in most cases hindered by the robustness of the sphere arrangement. In fact, artefacts are more prone to happen in disordered regions of the opal, in which the spheres have, on average, fewer interacting neighbours, and thus lower interparticle cohesion. Therefore, the choice of highly ordered opal regions was crucial for reliable AFM characterization. Moreover, the system needed to be stabilized in terms of water uptake/release under changing environmental conditions.

Besides, since we are interested in quantifying lattice features of the opal, a low degree of disorder in the opal is important as the centre-to-centre distance between particles increases respective to that of a perfect fcc packing. In practice, opals are always fabricated from particle suspensions with a certain polydispersity, which will determine some degree of disorder. In the case of opals made from silica particles, this polydispersity must be less than 5% to obtain opals of sufficient quality.^17^ In addition, the disorder in opals can vary randomly from one area of the sample to another depending on other factors that are much more difficult to control, such as the local quality of the substrate, the control of humidity, the temperature at which they are grown, or the presence of larger particles.^17^ At any rate, it is always possible to find areas of higher or lower order regardless of the quality of the starting particles. These aspects can be quantified by appropriate analysis of the AFM images, as shown in the next sections.

### B Analysis methodology

An opal straightforwardly provides a system with a large number of particle arrays with nearly identical configurations and periodicity, allowing reliable statistical analysis from the images. For our purposes, we need a proper tool that analyses geometric parameters (*e.g.*, the lattice parameter) in the real space and, simultaneously, assesses the quality of the particle arrangement to obtain robust quantitative information from the opal. Amongst different techniques, such as Fast Fourier Transform, we chose self-correlated imaging (SCI) since it gives a direct and fast measure of the periodicity in the sample.

We routinely analysed the AFM images with the open WSxM software^18^ using the following self-correlation function:

1 *G*(*k*_1_, *k*_2_) = Σ*f*(*x*, *y*) *f*(*x* + *k*_1_, *y* + *k*_2_)

where the function *f* denotes the height matrix measured in the AFM image. The equation correlates the image *f*(*x*, *y*) with any shifted image *f*(*x* + *k*_1_, *y* + *k*_2_), where *k*_1_ and *k*_2_ are any integer multiple of the pixel lengths along, respectively, the *X* and *Y* axes of the image. The resulting self-correlated image (SCI) *G*(*k*_1_, *k*_2_) is formed in the real space and it is non-zero whenever a correlation between the overlapped AFM images exists.

**Fig. 2:**
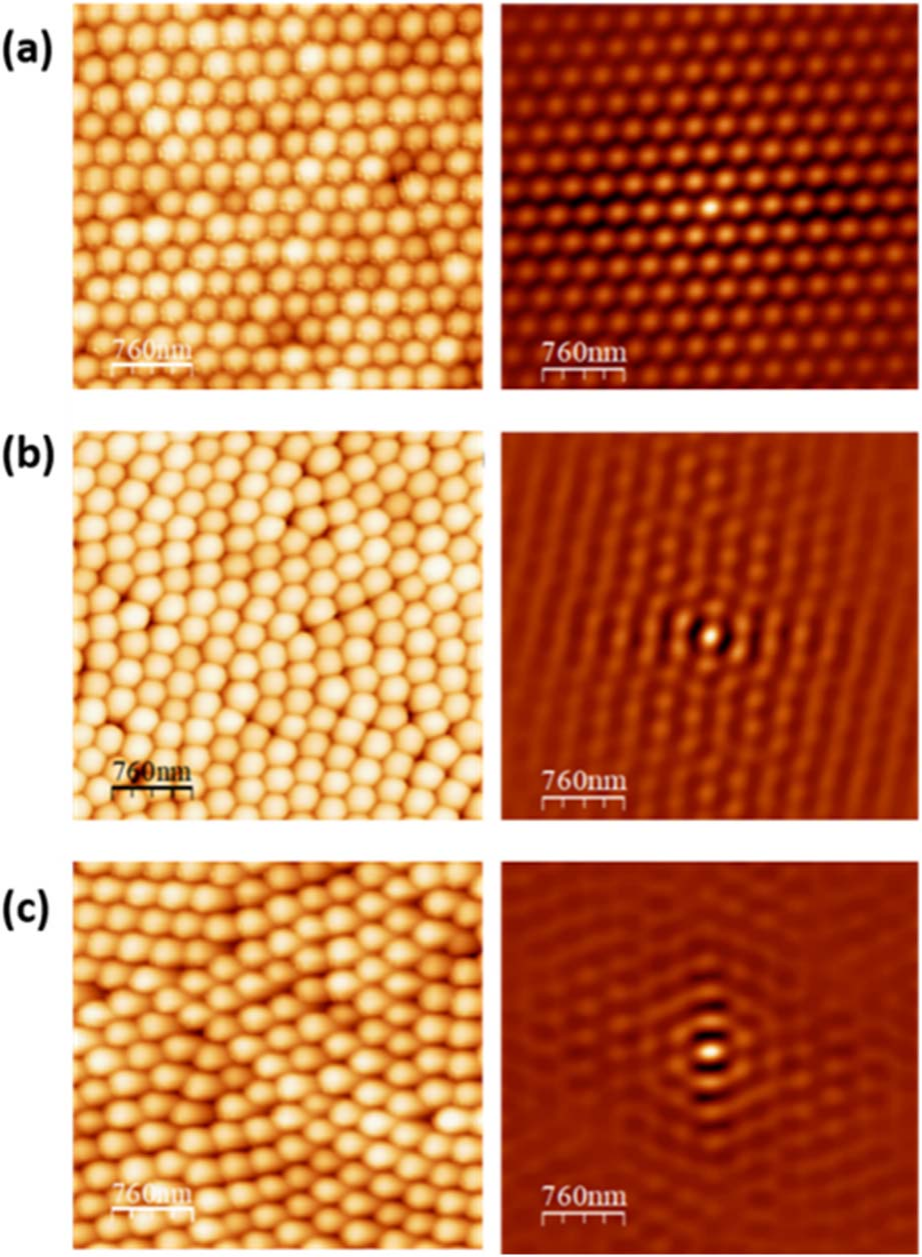
AFM images and self-correlated images (left and right columns, respectively) of the upper (111) plane of a 240 nm silica sphere opal with increasing disorder, (a–c). The calculated NSOP values were 0.67, 0.50 and 0.43, respectively. AFM experiments were performed in air (26 °C and 40% RH).

Here we propose the use of self-correlation functions on AFM images of opals in order to establish their ordering degree and geometric characteristics. Fig. 2 shows AFM images of different opal areas (left column) and the corresponding SCIs (right column). A highly ordered opal region yields, upon self-correlation, a nearly perfect hexagonal array of narrow dots (Fig. 2a). By contrast, a higher degree of disorder becomes straightaway evident in the SCIs: Fig. 2b and c show progressively irregular regions leading to increasingly distorted self-correlated patterns (broader dots, deformed hexagons). The self-correlation analysis also provides quantitative information on disorder by means of the normalized spatial order parameter (NSOP), calculated from the crossing centre profiles drawn across the vertexes of the central hexagon in the SCI according to2
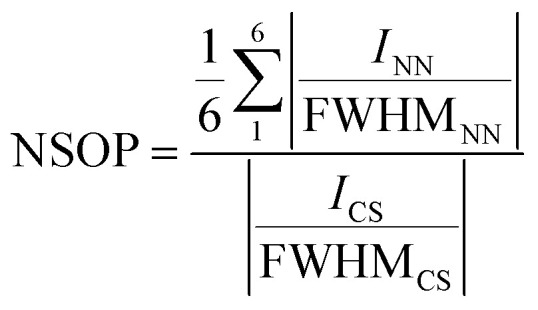
where *I*_CS_ denotes the height of the central spot of the SCI, and *I*_NN_ denotes the height of the nearest neighbouring spots.^19^ (Note that NSOP is defined this way to allow comparison between images with different numbers of pixels.) In the case of an ideally ordered opal area (perfectly hexagonal arrangement of identical spheres), NSOP = 1, and it decreases down to 0 by increasing opal disorder. In Fig. 2a–c, calculated NSOP values progressively decreased, consistently with the abovementioned statements. Finally, and as an important utility of the self-correlation analysis, the intervals between the NN and CS spots directly yields the average centre-to-centre distance (*Λ*) between the spheres in the imaged opal area, which is proportional to the (average) lattice parameter of the opal fcc packing in the selected region. The lattice parameter *d*_111_ refers to the spacing of the (111) planes. In an ideal close-packed fcc sphere assembly, *d*_111_ = (2/3)1/2*D*, where *D* is the sphere diameter. In our case, assuming close-packing, *D* = *Λ*.

### C Criterion for selection of opal areas

**Fig. 3:**
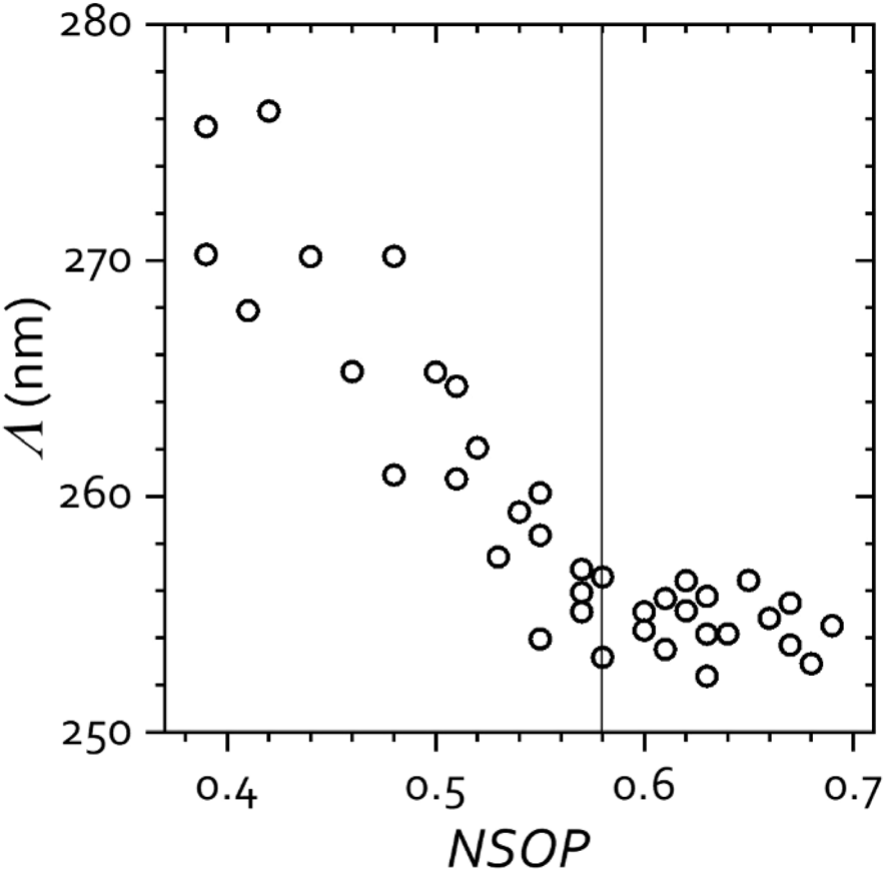
Behaviour of the centre-to-centre distance *Λ* as a function of the NSOP of self-correlated 1.3 × 1.3 μm^2^ images of a 240 nm silica opal. The dashed line denotes the threshold value of NSOP considered in this work for reliable quantification. AFM experiments were performed in air (26 °C and 40% RH).

The use of the self-correlation and the NSOP parameter provides us with an objective criterion to select AFM images for accurate measurement of the lattice parameter. As mentioned above, any opal disorder necessarily implies a deviation from the ideal fcc sphere packing, so that the calculated centre-to-centre distances *Λ* will be overrated to some extent (and larger value dispersion will be obtained). In order to delimit this source of error and establish an empirical rule for reliable measurements, we first investigated the dependence of *Λ* as a function of NSOP by analysing different opal areas with varying degree of disorder under identical conditions. Fig. 3 shows that *Λ* (and also its dispersion) clearly increased for lower NSOP, *i.e.*, for lesser order, but steadily decreased for higher NSOP (better order), tending to a constant value for NSOP > 0.58. The behaviour of the measured *Λ* certainly agrees with the expected trend, supporting the validity of the methodology.

In spite of the high quality of the opals employed, some degree of disorder is always present, so that, on one hand, the larger the surface area examined, the lower the order parameter. On the other hand, the larger the area, the more sphere arrays are evaluated and the better is the subsequent statistics. As a crossover between optimal order (smaller areas) and optimal statistics (larger areas), and on account of the behaviour exhibited by *Λ*, we find a convenient empirical rule to consider only 1.3 × 1.3 μm^2^ opal images with NSOP > 0.58. With this criterion, we obtain accurate measurements of *Λ* with an uncertainty of about 2 nm in opals of 240 nm silica spheres (<1%).

### D Assessment of lattice variation

**Fig. 4:**
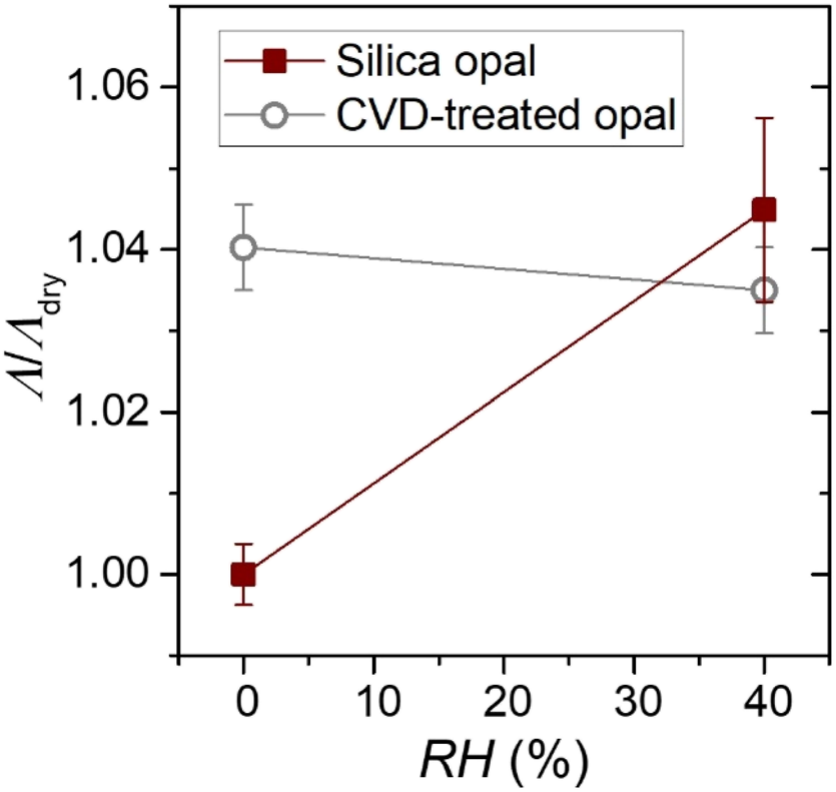
Centre-to-centre distance *Λ* from self-correlated AFM images of a silica opal (solid squares) and an opal treated by one-pulse-CVD (open circles). The samples were measured in ambient air (40% RH) and a dry nitrogen atmosphere (∼0% RH). *Λ* is normalized to the value obtained for the opals in a dry ambient. AFM experiments were performed at 26 °C.

As a proof-of-principle of our method, we primarily aim to directly assess whether the presence of moisture leads to any change in the lattice parameter of the opal. Therefore, we compare the centre-to-centre distance in the opal between ‘humid’ and ‘dry’ atmospheres, that is, upon water adsorption or not, respectively. To this purpose, the same opal spot is first AFM imaged under ambient humidity conditions (*ca.* 40% RH) and, next, in a nitrogen atmosphere or in high vacuum (under these conditions and prior to the measurement, the sample was heated at 67 °C to eliminate any water previously adsorbed). Fig. 4 shows how the average centre-to-centre distance *Λ* in air increased by about 4 ± 1% compared to dry conditions, and decreased back to its initial value after restoring the original humid conditions (*Λ* values were normalized in the figure). Since a silica opal in the air atmosphere contains about 8 wt% adsorbed water,^3^ this behaviour demonstrates that the lattice parameter reversibly increases in the presence of water. See ESI 2† for further experimental details related to AFM imaging under varying humidity conditions.

For comparison, we aimed at repeating the experiment on an opal with hindered ability to adsorb water, so that *Λ* would be expected to remain unchanged. To obtain such a sample we subjected a ‘wet’ opal (containing adsorbed water from the surrounding moisture) to one-pulse chemical vapor deposition (CVD),^20^ so that just the adsorbed water is transformed into silica without additional deposition. Thus, the treated opal remains practically identical, but the microporosity of the opal is mostly blocked by the deposited silica, largely preventing new water adsorption. Indeed, AFM measurements yielded no change (within the experimental error) of *Λ* in the treated opal from a humid to a dry atmosphere (Fig. 4). Note that we refrained from using annealing, which would also have hindered water adsorption,^5^ as it is a more aggressive procedure that induces significant disorder due to the strong thermal tensions. By contrast, NSOP values, which ranged from 0.62 to 0.65 in the normal opal, barely reduced after CVD treatment.

### E Temperature-dependent lattice behaviour

**Fig. 5:**
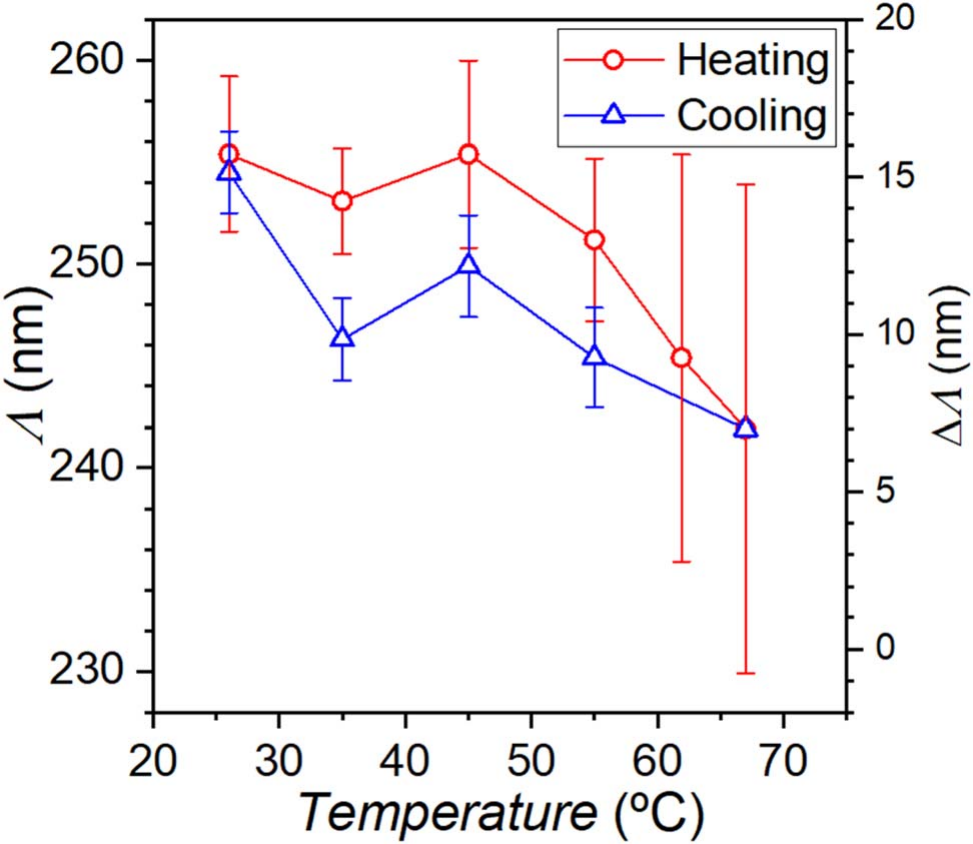
Evolution of the centre-to-centre distance *Λ* from self-correlated AFM images of a silica opal upon increasing (solid red circles) and posteriorly decreasing temperature (open blue triangles).

The precision of the experimental method further allows monitoring the opal behaviour upon progressive water desorption and re-adsorption. Therefore, we measured the evolution of *Λ* by AFM imaging the silica opal, in ambient air, upon increase and posterior decrease of the temperature. Note that although the mechanism of temperature-driven lattice variation differs from that caused by humidity changes, it provides valuable insight into the morphological transformations resulting from variations in water content. On the other hand, experimentally, temperature control offers higher precision, as it can be more locally adjusted. Fig. 5 demonstrates that the experiment indeed permitted clear distinguishing of an ongoing decrease in *Λ* of several nm towards higher temperatures, *i.e.*, lower water uptake, in accordance with the behaviour stated above. It is noteworthy that the measurement errors generally increased at higher temperatures, a fact that we tentatively attribute to the decreasing capillary cohesion between the opal spheres,^12^ leading to larger experimental noise, as discussed before. A clear hysteresis was also observable, so that *Λ* along water re-adsorption (cooling cycle) was somewhat smaller than during desorption (heating cycle). This performance would indicate that, at a given temperature, the opal retains more water (and, so, *Λ* is larger) upon desorption than re-adsorption, a conclusion that agrees with the hysteretic behaviour of water adsorption isotherms measured in dense ensembles made with similar silica spheres.^22^

## Discussion

Fig. 4 directly assesses the increment of a silica opal lattice in a humid ambient, and confirms that our experimental procedure enables measurement of small but unambiguous variations in the opal morphology by changing *in situ* the ambient conditions. Quantitatively, Fig. 4 and 5 demonstrate a significant increase of the opal lattice parameter under moist conditions (humid air at room temperature) up to 4–5% respective to vacuum conditions. It must be assumed that, in the latter case, the silica spheres still contain some chemisorbed water partially filling their micropores. Note that while physisorbed water can easily be evaporated at low temperature, chemisorbed water, held by strong chemical bonds, requires temperatures exceeding 400 °C for removal.^3^ The extent of the lattice increment in our opal, of several nanometres, is consistent with previous estimates obtained from PBG measurements.^4^ This quantification is possible because our particle system undergoes uniform swelling that preserves lattice order under all tested humidity and temperature conditions. However, it must be noted that this AFM method only explores the upper (111) plane, whose variation may somewhat differ in magnitude from that of the bulk opal. This aspect will be subject of future research.

Finally, one must take into account that the accuracy of AFM techniques (or other imaging techniques, such as scanning electron microscopy (SEM)) barely allows discerning changes in individual spheres but variations averaged over arrays of several to many spheres. Thus, regarding the phenomenon which ultimately causes the lattice increase upon water adsorption, we cannot conclude an increment of the diameter of single particles from direct observation. However, recent isothermal studies have definitely proved the disputed existence of significant microporosity in Stöber spheres accessible to water molecules.^3^ More specifically, an original isothermal analysis has shown that the internal volume of Stöber spheres increased during water filling of the microporosity,^21^ which was attributed to adsorption-induced swelling of the silica backbone. Furthermore, sphere swelling upon water adsorption has also been deduced from novel fitting of PBG properties.^6^ Thus, in view of these recent findings, we attribute the changes in the lattice parameter upon water uptake to swelling of the Stöber silica spheres. In any case it must be taken into account that swelling is strongly dependent on microporosity, which in turn greatly depends on synthesis conditions, aging, *etc.*, so there might be quantitative discrepancies between different silica opals. Additionally, as microporosity changes with the distance within the particle, the inner part is more porous than the outer part, and there might be variations in the swelling behaviour depending on the particle size, which remains an open topic for future investigations.

Although AFM measurements inherently probe only the surface of the opal, the observed lattice expansion should not be interpreted as a purely two-dimensional effect. Our previous optical studies provide compelling evidence that these lattice parameter changes extend throughout the entire three-dimensional photonic crystal. The Bragg peak shift observed in optical experiments reflects structural modifications occurring across the full opal lattice, confirming that the expansion is not restricted to the uppermost layers. In fact, part of the optical properties, particularly the first order Bragg peak, respond exclusively to the lattice periodicity in the normal direction. In consequence, the Bragg peak alone reveals the lattice parameter in the vertical direction from a macroscopic area and to a depth of about the Bragg length.The strong agreement between our AFM measurements and prior optical studies robustly supports the conclusion that the lattice expansion is a bulk phenomenon affecting the entire opal. This consistency underscores the validity of AFM as a precise tool for probing nanoscale lattice variations in self-assembled colloidal systems.

Summarizing, this study reveals nanoscale size variations in colloidal silica spheres using AFM for the first time, detecting changes of just a few nanometers with high precision. This advancement was achieved in highly ordered arrangement of the particles, which enabled the use of autocorrelation techniques to precisely detect such nanometric variations. The research enhances understanding of colloidal silica, a multifunctional material widely used in photonics, catalysis, and environmental science. These findings hold significance for both fundamental studies and technological applications.

## Conclusions

We present an innovative procedure to utilize AFM for the study the morphology of colloidal crystals. We use self-correlated imaging, obtained from the AFM images, that provides a precise quantification of the lattice parameters, profiting from statistical analysis of many-particle arrangements, and a robust criterion for selection of suitable measuring spots based on an order parameter. An accuracy of less than 1% in centre-to-centre distances is achieved.

A significant, reversible increment of the lattice parameter of our silica opals in about 4% is directly assessed by *in situ* AFM imaging of the sample under varying humidity and temperature, which we attribute to silica swelling upon water filling of the sphere micropores. This direct observation (by contrast with previous reports based on rather complex, deductive methods) is a relevant finding, as it unambiguously proves that lattice expansion of sphere assemblies can occur upon vapour sorption. This procedure not only overcomes the limitations of indirect measurement methods (such as fitting of photonic parameters), but also allows the *in situ* application of AFM for detailed exploration of colloidal crystals and their response to changing environmental conditions. This latter aspect is essential, for instance, for the design of photonic materials, where the optical properties depend significantly on the structure of the material and its environment.

## Experimental

### Sample preparation

Monodisperse colloidal suspension, comprising Stöber silica spheres (Microparticles GmbH, nominal polydispersity of <3%, approximate diameter of 240 nm as measured by SEM), is diluted in ethanol with a 2% weight/weight concentration. The vertical deposition technique, undertaken within a Binder KBF constant climate chamber set at 50 °C and 60% humidity, facilitates the formation of the samples. Prior to deposition, both the glass substrate and vial undergo a 24 hour hydrophilization process in hydrochloric acid. This assembly, along with the substrate, is then thermostatically conditioned in the climatic chamber for 20 minutes. The substrate is carefully introduced into the vial at an angle ranging from 0 to 5° relative to the vertical. Post evaporation of the solvent, a face-centred cubic structure of silica spheres is achieved. The final opals, roughly 1 cm^2^ in size, undergo rigorous quality assessment through optical characterization and SEM.

### Atomic force microscopy

Non-contact dynamic AFM experiments were carried out to scan the upper (111) plane of the opal sample in an environmental chamber that allowed measurement in air (with typical relative humidity RH of ∼40%) in a nitrogen atmosphere (>99 999% purity, ∼0% RH), or even in high vacuum. Additionally, the sample temperature was increased *in situ* with a heater to further control the water adsorption during the experiment.

Two different Cervantes Full mode AFM systems from Nanotec Electronica SL, together with PPP-FMR probes from Nanosensors, were used for the measurements. We employed WSxM software (http://www.wsxm.es/) both for data acquisition and image processing.^18^ One of the systems is enclosed in an adiabatic box with a nitrogen flow pipeline and a hygrometer to control humidity. The other system is mounted into a high vacuum chamber. For measurements with temperature, the sample is mounted onto a hot plate connected to a resistance, where current passes and heats the sample. The AFM measurements were carried out upon reaching thermal stabilization, with 30 minutes of waiting time.

AFM measurements were performed in dynamic mode, with a typical oscillation amplitude of 15 nm. In most cases, the phase locked loop (PLL) was enabled to track the resonance frequency. In order to ensure stable scanning of the samples under the different conditions, we used non-contact amplitude modulation mode for ambient conditions and drive amplitude modulation mode^22^ for HV measurements. We employed PPP-FMR cantilevers from Nanosensors (http://www.nanosensors.com/), with a nominal resonance frequency of 75 kHz and spring constant of 2.8 N m^−1^.

## Data availability

Data for this article are available at DIGITAL.CSIC repository at https://digital.csic.es/.

## Conflicts of interest

There are no conflicts to declare.

## Supplementary Material

NA-OLF-D5NA00127G-s001
