# Supplementary material for: Lattice variation upon water adsorption in silica opals measured by in situ atomic force microscopy
Source: Nanoscale Adv. 2025 Mar 21;7(10):3049–54. doi: 10.1039/d5na00127g (PMC11969375; doi:10.1039/d5na00127g)
Supplement: NA-007-D5NA00127G-s001 [file NA-007-D5NA00127G-s001.pdf]

Supplementary Information (SI) for Nanoscale Advances.
This journal is © The Royal Society of Chemistry 2025

SUPPORTING INFORMATION

Lattice variation upon water adsorption in silica opals measured by in situ atomic force microscopy

Francisco Gallego-Gómez, Eider Berganza, Miguel Morales, Álvaro Blanco, Cefe López, Agustina Asenjo and Miriam Jaafar.

1 SILICA OPAL DIAMETER QUANTIFICATION VIA SCANNING ELECTRON MICROSCOPY

The diameter of the studied silicon opal sample was determined by Scanning Electron Microscopy (SEM). A representative image is displayed on Figure S1.

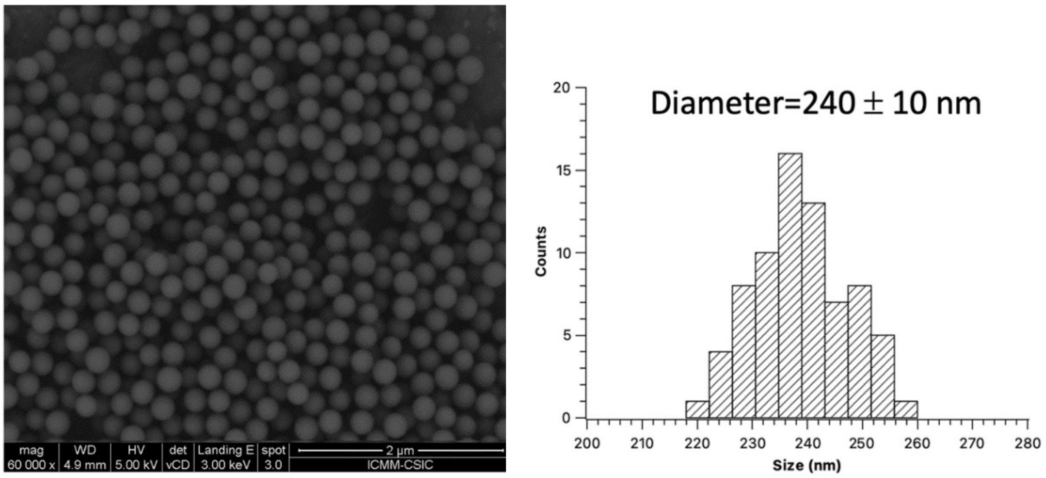

Figure S1. SEM images of the studied opal and measured average diameter

2 AFM IMAGING IN VACUUM CONDITIONS

Silica opal measured at different relative humidity conditions. Generally, images measured at 40% or ambient humidity conditions show good stability, while at 0% RH (vacuum conditions), the cohesion between opals is compromised, which yields instabilities which are translated to the images (see Figure S2).

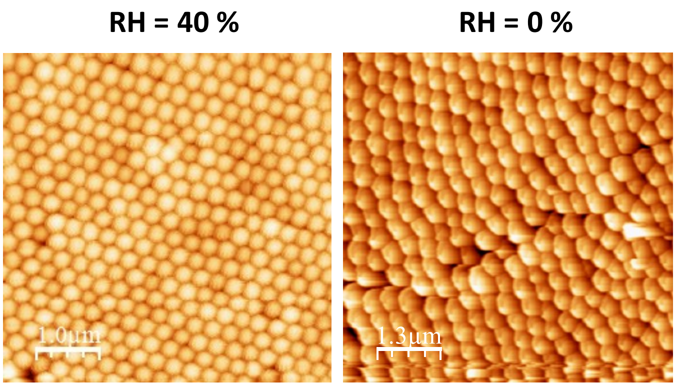

Figure S2. Representative images of the silica opal acquired at 40% and 0% relative humidity conditions.
